# News and Notes

**Published:** 1906-05

**Authors:** 


### *NEWS AND NOTES.*

DR. E. MORGAN died at his home in Arlington, Ga., April 10th.

DR. PAUL McDONALD has moved from Gwinnett county to Bolton, Ga.

DR. J. S. TODD, while riding up to his residence, was thrown from his horse April the 12th, and sustained a fracture of his collar-bone.

THE Georgia State Board of Regular Medical Examiners was to hold examinations for license to practice medicine and surgery in the State of Georgia, at Augusta, on April 3d and 4th, and at Atlanta on April 5th and 6th.

DECATUR COUNTY MEDICAL SOCIETY.—This society was recently organized at Bainbridge, on the standard plan. Dr. Vega Berry was elected president; Dr. Gordon Chason, vice-president, and Dr. Henry H. Cheatham, secretary and treasurer, all of Bainbridge.

THE physicians of Hancock county met February 27, with Dr. W. W. Pilcher, Warrenton, councilor for the tenth district, at Sparta, and organized this society on the standard plan. The following officers were elected: President, Dr. R. C. Wiley, Sparta; vice-president, Dr. R. G. Stone, Linton; secretary and treasurer, Dr. James G. Harrison, Sparta; censors, Dr. R. G. Pattillo, Culverton; Horace R. Darden, Sparta, and R. L. Ray, and delegate to the State Association, Dr. Alexander F. Durham, Sparta.

THE thirty-second annual report of the medical director of the Cincinnati Sanitorium has just been issued, and makes a splendid showing for this well-known private hospital for persons afflicted with nervous and mental disorders.

The "daily average" of inmates was the highest attained in the history of the institution. Important additions to the medical equipment have been made during the year, including a new examination and operating-room and a modern clinical laboratory. The filtering system of the general water-supply has been augmented, and the electric lighting has been extended throughout the grounds, amusement halls and other portions of the establishment. Being in the suburbs of Cincinnati, its opportunities for amusements, shopping, services of skilled specialists in the various branches of medicine, etc., are distinctly advantageous to privileged patients. With an excellent medical staff, and many other advantages, we can easily account for the continued prosperity of the Cincinnati Sanatorium.

---

THE commencement exercises of the Atlanta School of Medicine were held at the Grand Opera House April the 3d. Dr. George H. Noble, the dean, reported that two hundred and twenty students had been matriculated during the first term, which was considered unusually encouraging for a college not yet a year old.

Gov. W. J. Northen, president of the board of trustees, delivered the diplomas to twenty-one graduates. Hon. Hooper Alexander made the annual oration, and Bishop C. K. Nelson gave a benedictory address.

The faculty is now constructing college buildings on Luckie, Bartow and Cain streets, at a cost of $65,000, to be ready for the next term.

The graduates are J. F. Booth, Louisiana; S. R. Brown, Alabama; J. A. Davis, Florida; H. C. Ellis, Georgia; R. T. Foster, Texas; G. T. Hendry, Georgia; P. F. Lisk, Florida; G. R. Luke, Georgia; A. W. Lane, Alabama; G. C. Lyon, Louisiana; W. L. McManus, Georgia; E. L. Norton, Georgia; W. W.

Puett, Georgia; O. W. Roberts, Georgia; H. Rushin, Georgia; D. S. Reese, Georgia; G. S. Sumner, Georgia; J. C. Vinson, Georgia; R. E. Wilson, Georgia; M. Ward, Georgia, and W. E. Yankey, Georgia.

---

AT the commencement exercises of the Atlanta College of Physicians and Surgeons, April 2d, it was announced that Andrew Carnegie and Dr. A. W. Calhoun had each given $10,000 to the college. In consideration of these generous gifts the faculty and board of trustees decided to establish the Andrew Carnegie Library of Pathology and the A. W. Calhoun Chair of Opthalmology.

The corner-stone of the new college building was laid Friday, April the 6th, and $80,000 has been subscribed by the faculty and citizens of Atlanta for its completion. They expect to have it ready for use at the coming term of college. Fifty-two were graduated in medicine, their diplomas being delivered by Judge T. P. Westmoreland.

The honor graduates announced by Dr. Elkin are T. C. Davison, Cleveland Davis, F. M. Ridley, Jr., W. P. Ellis, G. G. Stribling, F. Schnauss, E. A. Beech and A. P. Manget, while the hospital appointments are E. A. Boeckh, F. P. Manget and G. B. Nunnally.

The graduates in medicine are S. W. Anthony, G. D. Ayer, G. Z. Baber, E. A. Boeckh, W. H. Chapman, E. Collum, J. T. Dabbs, Cleveland Davis, T. C. Davidson, O. J. DeLoach, W. J. Dickson, J. C. Ellis, W. P. Ellis, E. D. Ford, J. O. Foster, J. A. Garrett, R. B. Gilbert, W. Y. Gilliam, O. D. Hall, A. S. Hargrove, E. D. Highsmith, J. H. Knox, R. B. Lamb, W. E. Lee, H. G. Lightner, W. H. Lott, F. P. Manget, W. L. Marshall, C. B. Mashburn, O. H. Matthews, G. M. Mitchell, D. E. Morgan, G. C. McKenzie, H. B. Nunnally, J. A. Otwell, H. H. Parr, Codson Reed, F. M. Ridley, Jr., W. R. Roberts, E. A. Russell, F. W. Schnauss, B. T. Smith, G. B. Stribling, J. O. Teasley, Rufus Thames, J. W. Watson, C. B. Welch, J. B. Welden, G. M. White, G. O. Williams, A. J. Williams and C. H. Winn.

